# First person – Marie Rodinova

**DOI:** 10.1242/dmm.041285

**Published:** 2019-07-26

## Abstract

First Person is a series of interviews with the first authors of a selection of papers published in Disease Models & Mechanisms, helping early-career researchers promote themselves alongside their papers. Marie Vanisova (née Rodinova) is first author on ‘Skeletal muscle in an early manifest transgenic minipig model of Huntington's disease revealed deterioration of mitochondrial bioenergetics and ultrastructure impairment’, published in DMM. Marie is a PhD student in the lab of Hana Hansikova in the Department of Paediatrics and Adolescent Medicine, Charles University and General University Hospital in Prague, Czech Republic, investigating mitochondrial dysfunction in Huntington's disease (HD) and its role in the progress of HD.


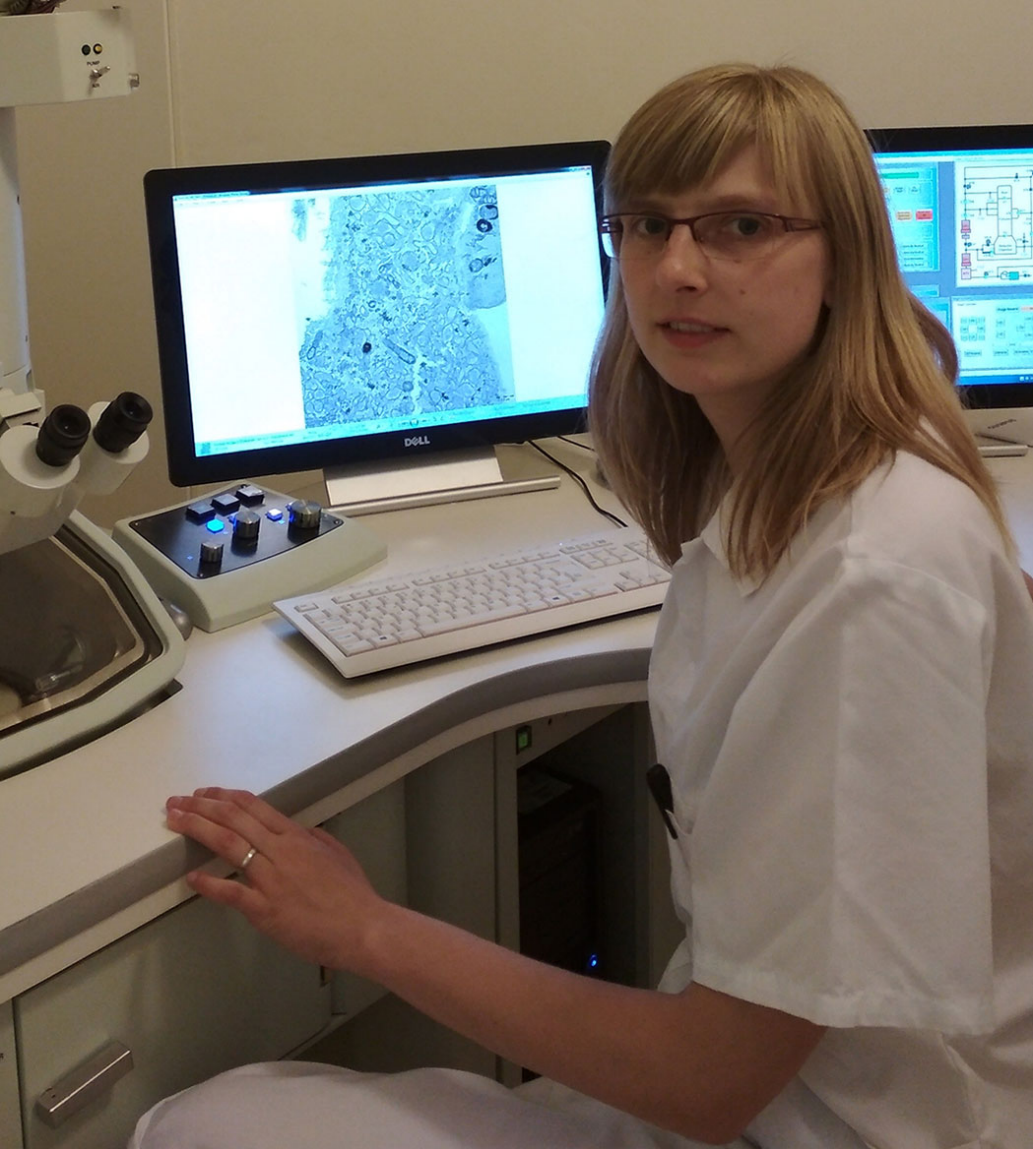


**Marie Vanisova (née Rodinova)**

**How would you explain the main findings of your paper to non-scientific family and friends?**

Huntington's disease (HD) is a rare progressive neurodegenerative disease affecting mostly adults and presents with movement difficulties, personality changes, weight loss and dementia, usually leading to death 15-20 years after disease onset. Muscular dysfunction significantly worsens a HD patient's quality of life, therefore, we have focused on the characterization of skeletal muscle in HD. For this purpose we chose to use the minipig animal model for HD (TgHD minipigs). As skeletal muscle has a high energy demand, we focused especially on the mitochondrial metabolism, because mitochondria are essential organelles working alongside other features as cellular powerhouses. Moreover, mitochondrial damage is considered to be an important player in disease progression. We demonstrated early biochemical and structural changes in TgHD minipig muscle before the clinical symptoms occur. Decreased quality of mitochondria and impaired mitochondrial energy-producing pathway (OXPHOS system) were found in TgHD muscle compared with age-matched healthy controls.

**What are the potential implications of these results for your field of research?**

Our results are important for understanding the disease progression in different tissues and help us compile a timeline that describes how tissues (such as skeletal muscle, in our case) and their functions are affected. Working with the animal model gives us important information about the course of the disease without burdening HD patients. Our results could help in development of appropriate treatment to alleviate HD symptoms.

**What are the main advantages and drawbacks of the model system you have used as it relates to the disease you are investigating?**

The most important advantage of our minipig model is its similarity to the human disease, in contrast to mice or other small model animals. Minipigs have a long lifespan, which enables us to study the preclinical and clinical period. The disease progression is slow (as in patients), the brain has a similar anatomy to that of humans, the immune system works similarly and, thanks to the TgHD minipig's size, we can obtain sufficient amounts of biological material. On the other hand, relatively long life and the size of the minipig model poses a problem with time management of experiments and the space needed to house the animals. It is not surprising that using a large animal model is very expensive.

“The most important advantage of our minipig model is its similarity to the human disease, in contrast to mice or other small model animals.”

**What has surprised you the most while conducting your research?**

When I started doing the HD research, I knew some basic information about the impact of HD on the brain, but it is incredible how much the mutation of the huntingtin protein affects cellular processes in different tissues, and not only at different time points of disease progression, but also in different cellular compartments, especially mitochondria.

**Describe what you think is the most significant challenge impacting your research at this time and how will this be addressed over the next 10 years?**

Results obtained from muscle give us new directions and possibilities of research that can be applied in cardiac or neural tissue. Now, we can study HD in these tissues more deeply and efficiently and it could help us gain a better understanding of pathology.

**Figure:**
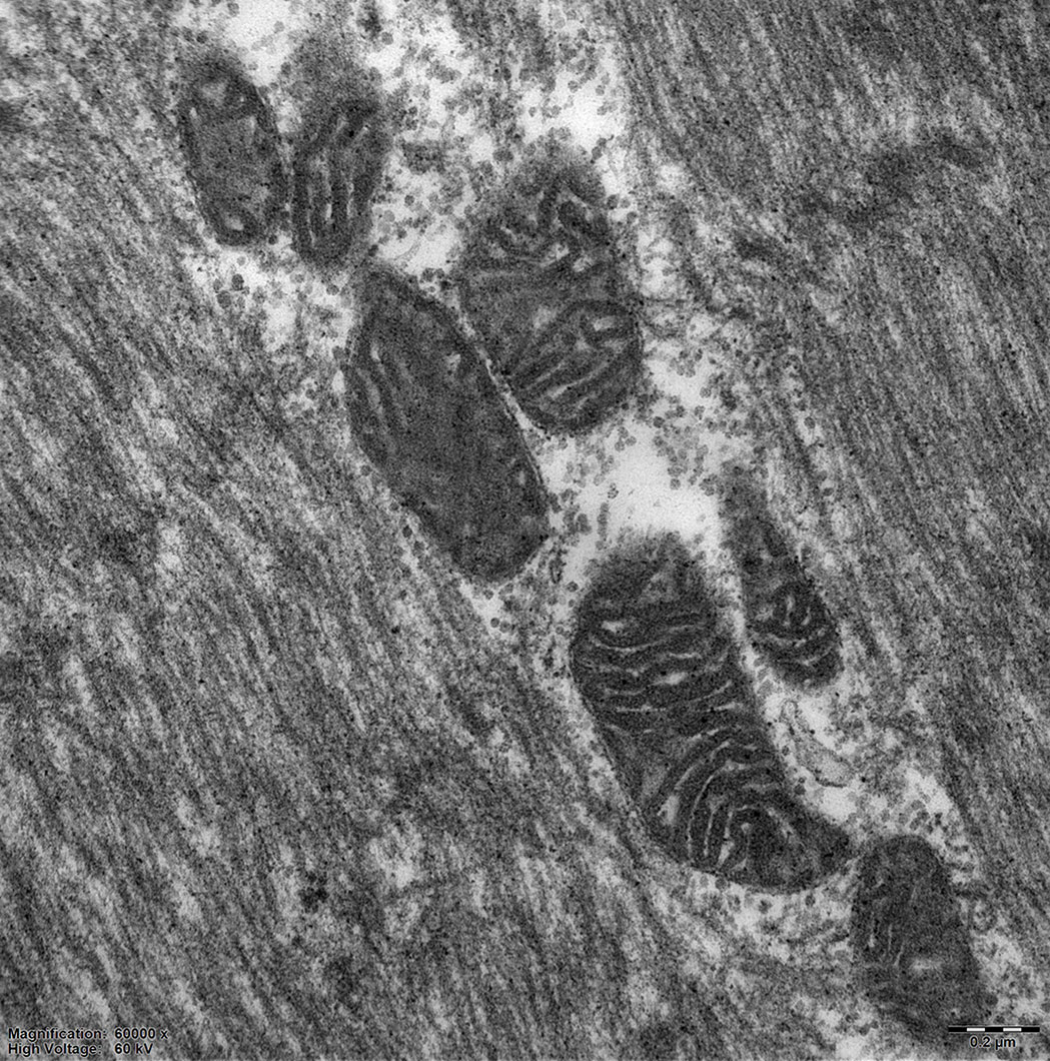
**Ultrastructure of the mitochondria from the WT minipig skeletal muscle at the age of 48 months obtained using a transmission electron microscope; original magnification: ×60,000.**

**What changes do you think could improve the professional lives of early-career scientists?**

Cross-disciplinary cooperation with young and also with experienced scientists, because it is important that the findings of basic research are translated and applied in practice. And thanks to my personal experience as a mother, I think it is very important to support young scientists who are also new parents and help them combine child care with the possibility to continue doing research.

“I think it is very important to support young scientists who are also new parents and help them combine child care with the possibility to continue doing research.”

**What's next for you?**

I am now returning to work after my maternity leave, so I am full of enthusiasm to get back into HD research with full commitment. Because my current time options are limited, I am learning to work more efficiently and with more focus than before, which is also a new challenge.
